# Synthesis, crystal structure and Hirshfeld surface analysis of 1-[(1-octyl-1*H*-1,2,3-triazol-4-yl)methyl]-3-phenyl-1,2-di­hydro­quinoxalin-2(1*H*)-one

**DOI:** 10.1107/S2056989024007746

**Published:** 2024-08-09

**Authors:** Nadeem Abad, Joel T. Mague, Camille Kalonji Mubengayi, Abdulsalam Alsubari, El Mokhtar Essassi, Youssef Ramli

**Affiliations:** a Laboratory of Medicinal Chemistry Drug Sciences Research Center Faculty of Medicine and Pharmacy Mohammed V University in Rabat Morocco; b Laboratory of Heterocyclic Organic Chemistry, Faculty of Sciences, Mohammed V University, Rabat, Morocco; c Department of Chemistry, Tulane University, New Orleans, LA 70118, USA; d Laboratoire de Chimie et Biochimie, Institut Superieur des Techniques Medicales de Kinshasa, Republique Democratique du , Congo; e Laboratory of Medicinal Chemistry, Faculty of Clinical Pharmacy, 21 September University, Yemen; Katholieke Universiteit Leuven, Belgium

**Keywords:** crystal structure, di­hydro­quinoxaline, triazole, hydrogen bond, C—H⋯π(ring) inter­action

## Abstract

In the title mol­ecule, C_25_H_29_N_5_O, the di­hydro­quinoxaline unit is not quite planar (r.m.s. deviation = 0.030 Å) as there is a dihedral angle of 2.69 (3)° between the mean planes of the constituent rings and the mol­ecule adopts a hairpin conformation. In the crystal, the polar portions of the mol­ecules are associated through C—H⋯O and C—H⋯N hydrogen bonds and C—H⋯π(ring) and C=O⋯π(ring) inter­actions, forming thick layers parallel to the *bc* plane and with the *n*-octyl groups on the outside surfaces.

## Abstract 2

The di­hydro­quinoxaline unit in the title mol­ecule is not quite planar and the mol­ecule adopts a hairpin conformation due in part to an intra­molecular C—H⋯O hydrogen bond. In the crystal, the polar portions of the mol­ecules are associated through C—H⋯O and C—H⋯N hydrogen bonds and C—H⋯π(ring) and C= O⋯π(ring) inter­actions, forming thick layers parallel to the *bc* plane and with the *n*-octyl groups on the outside surfaces.

## 1. Chemical context

**Figure 1:**
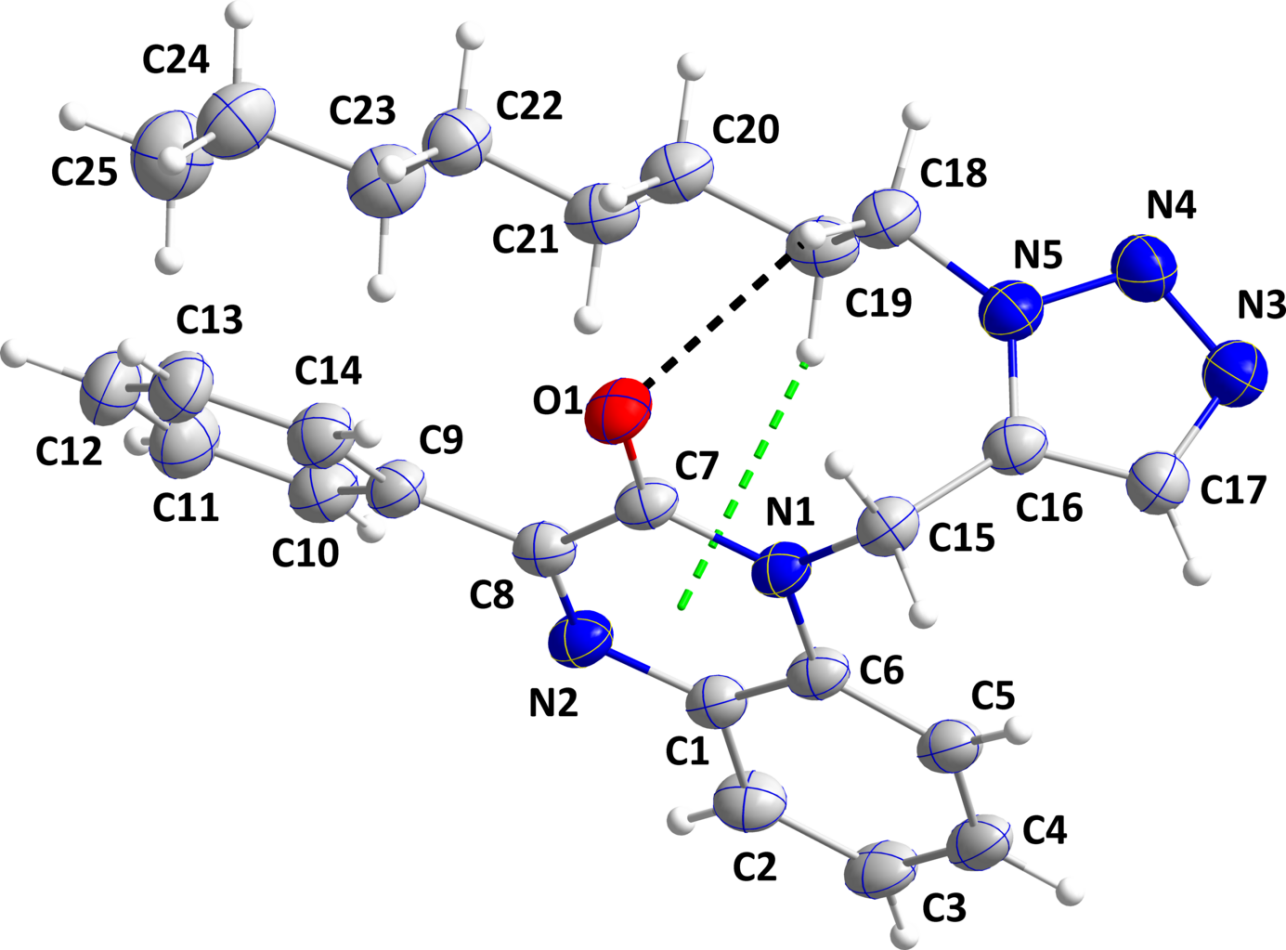
The title mol­ecule with labeling scheme and 50% probability ellipsoids. The intra­molecular hydrogen bond and C—H⋯π(ring) inter­action are shown, respectively, by black and green dashed lines.

The quinoxaline moiety is well known as a versatile nitro­gen-containing heterocyclic scaffold owing to its extensive pharmacological and biological properties as well as numerous therapeutic applications in medicinal research. It is reported to exhibit anti-tuberculosis (Carta *et al.*, 2001 ▸), anti-fungal (Wagle *et al.*, 2008 ▸), anti-HIV (Balzarini *et al.*, 2000 ▸), anti-microbial (Singh *et al.*, 2010 ▸), anti-malarial (Hui *et al.*, 2006 ▸), anti-cancer (Gupta *et al.*, 2005 ▸) and anti-inflammatory (Carta *et al.*, 2006 ▸) activities. Furthermore, quinoxalines possess anti­corrosion characteristics (*e.g.* Lgaz *et al.*, 2015 ▸). Similarly, the triazole ring system is linked to biological and pharmacological activities such as anti-fungal (Nowaczyk & Modzelewska-Banachiewicz, 2008 ▸), anti-bacterial (Foroumadi *et al.*, 2003 ▸), anti-hypertensive (Sato *et al.*, 1980 ▸), anti-Alzheimer’s disease (Missioui *et al.*, 2022*a* ▸), anti-COVID-19 (Zhang *et al.*, 2020 ▸) and anti­cancer (Shivarama *et al.*, 2003 ▸) activities. Given the wide range of therapeutic applications for quinoxaline and triazole derivatives, and with our continuing inter­est in the synthesis of heterocyclic systems having biological potential, we previously reported a route for the preparation of hybrid quinoxaline-containing triazoles (Missioui *et al.*, 2022*b* ▸) and herein report the synthesis and spectroscopic characterization of the new hybrid quinoxaline, 1-[(1-octyl-1*H*-1,2,3-triazol-4-yl)meth­yl]-3-phenyl-1,2-di­hydro­quinoxalin-2(1*H*)-one. A colorless plate-like specimen of the title compound was used for the X-ray crystallographic analysis (Fig. 1 ▸). A Hirshfeld surface analysis was performed to analyze the inter­molecular inter­actions.
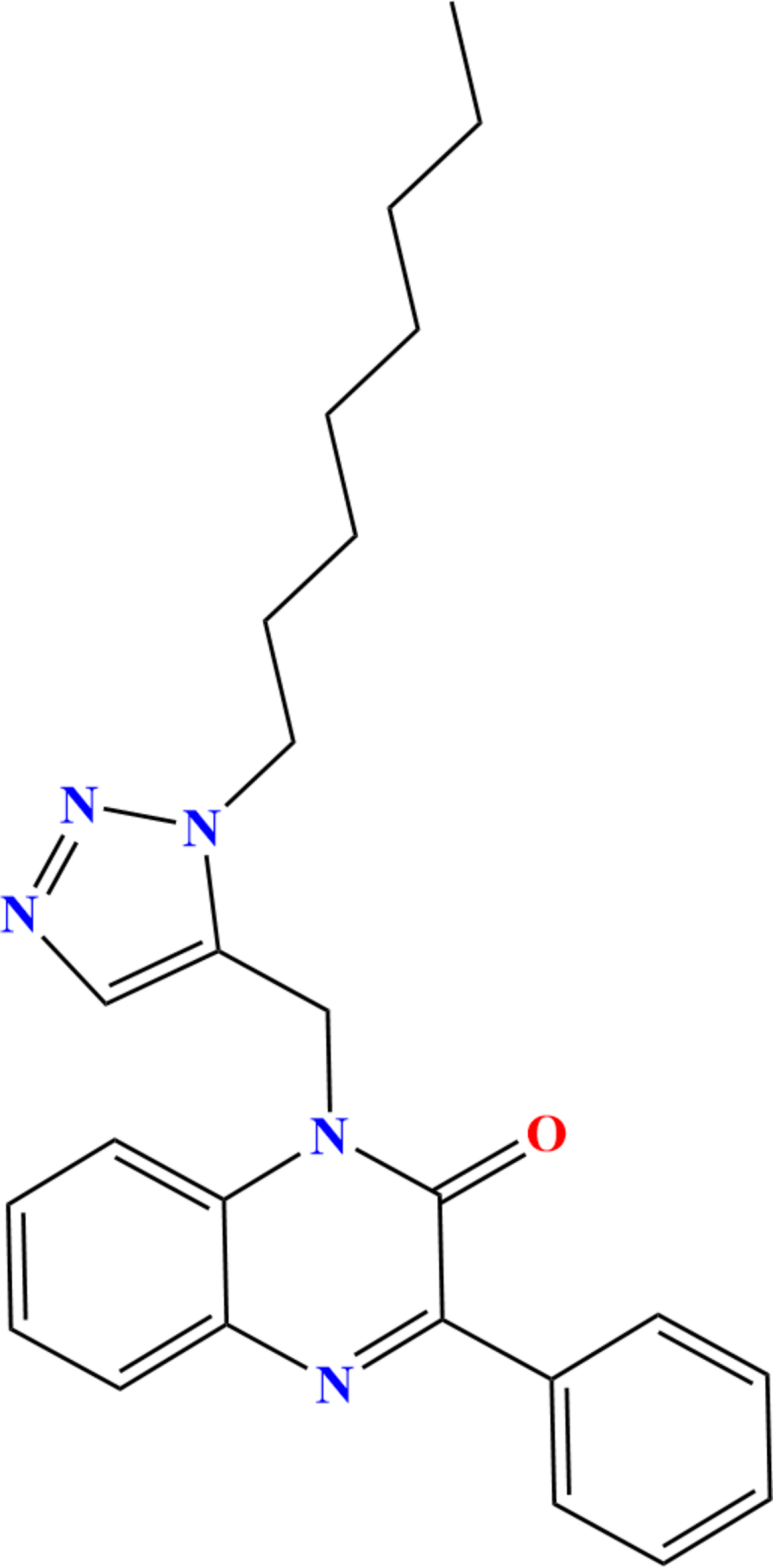


## 2. Structural commentary

The title mol­ecule adopts a hairpin conformation, in part due to an intra­molecular C18—H18*B*⋯O1 hydrogen bond and an intra­molecular C—H⋯π(ring) inter­action between C19—H19*A* and the C1/C6/N1/C7/C8/N2 ring (Table 1 ▸ and Fig. 1 ▸). The di­hydro­quinoxaline unit is not quite planar (r.m.s. deviation = 0.030 Å), as indicated by the dihedral angle of 2.69 (3)° between the constituent rings and by N1 being 0.044 (3) Å and C5 − 0.043 (3) Å from the mean plane of the ten-atom unit. The C9–C14 benzene ring is inclined to the mean plane of the C1/C6/N1/C7/C8/N2 ring by 24.0 (1)° while the C16/C17/N3/N4/N5 ring is inclined to the mean plane of the di­hydro­quinoxaline unit by 81.4 (1)°. The *n*-octyl chain is largely in the all-*trans* conformation, except for the portion closest to N5. Thus the N5—C18—C19—C20 and C18—C19—C20—C21 torsion angles are 169.2 (4) and −172.9 (4)°, respectively, while the remainder towards the terminus of the chain are in the range 175.0 (4)–179.8 (4)°.

## 3. Supra­molecular features

**Figure 2:**
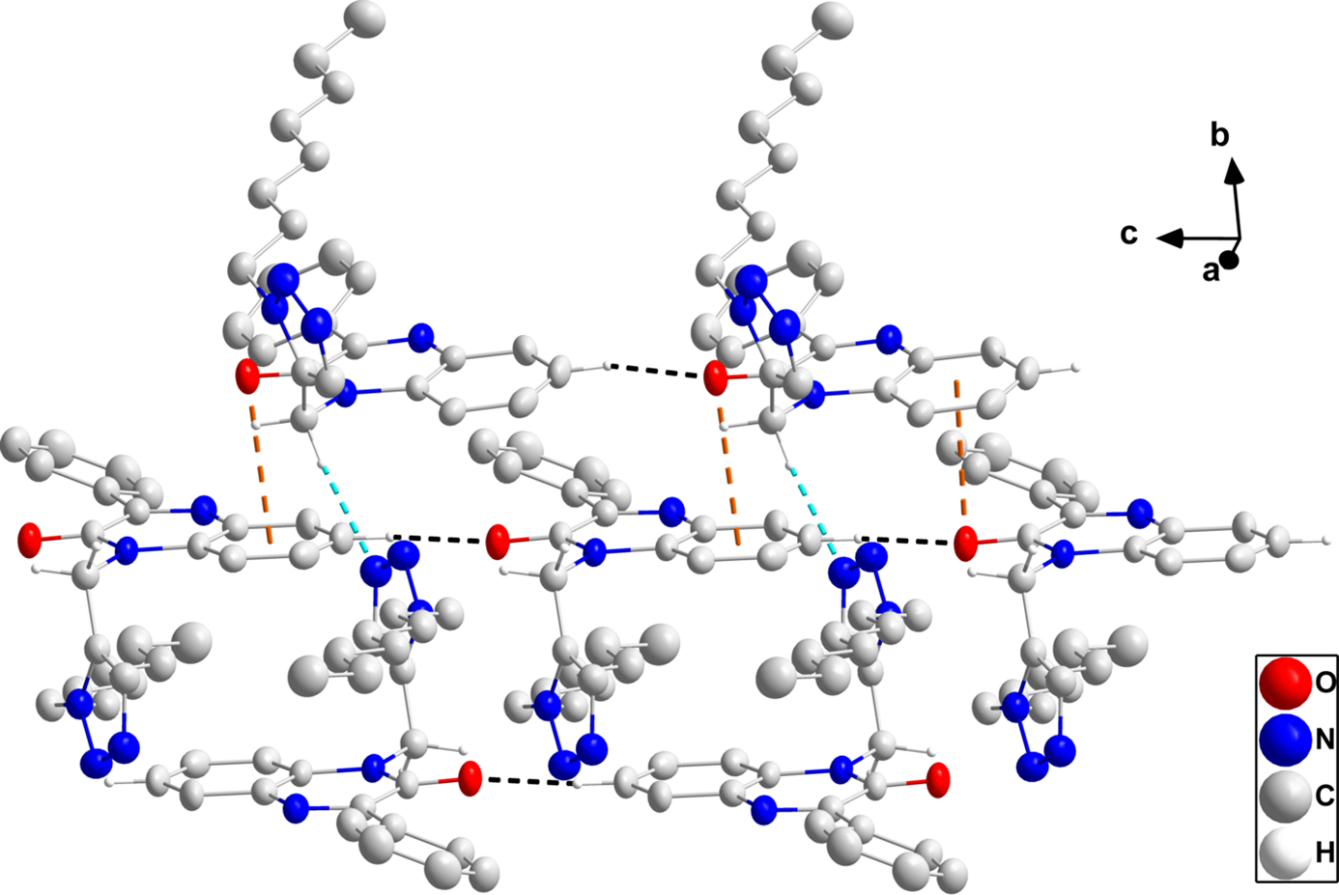
Detail of the inter­molecular C—H⋯N and C—H⋯O hydrogen bonds, which are shown, respectively, by black and light-blue dashed lines and the C=O⋯π(ring) inter­actions, which are shown by orange dashed lines. Non-inter­acting hydrogen atoms are omitted for clarity.

**Figure 3:**
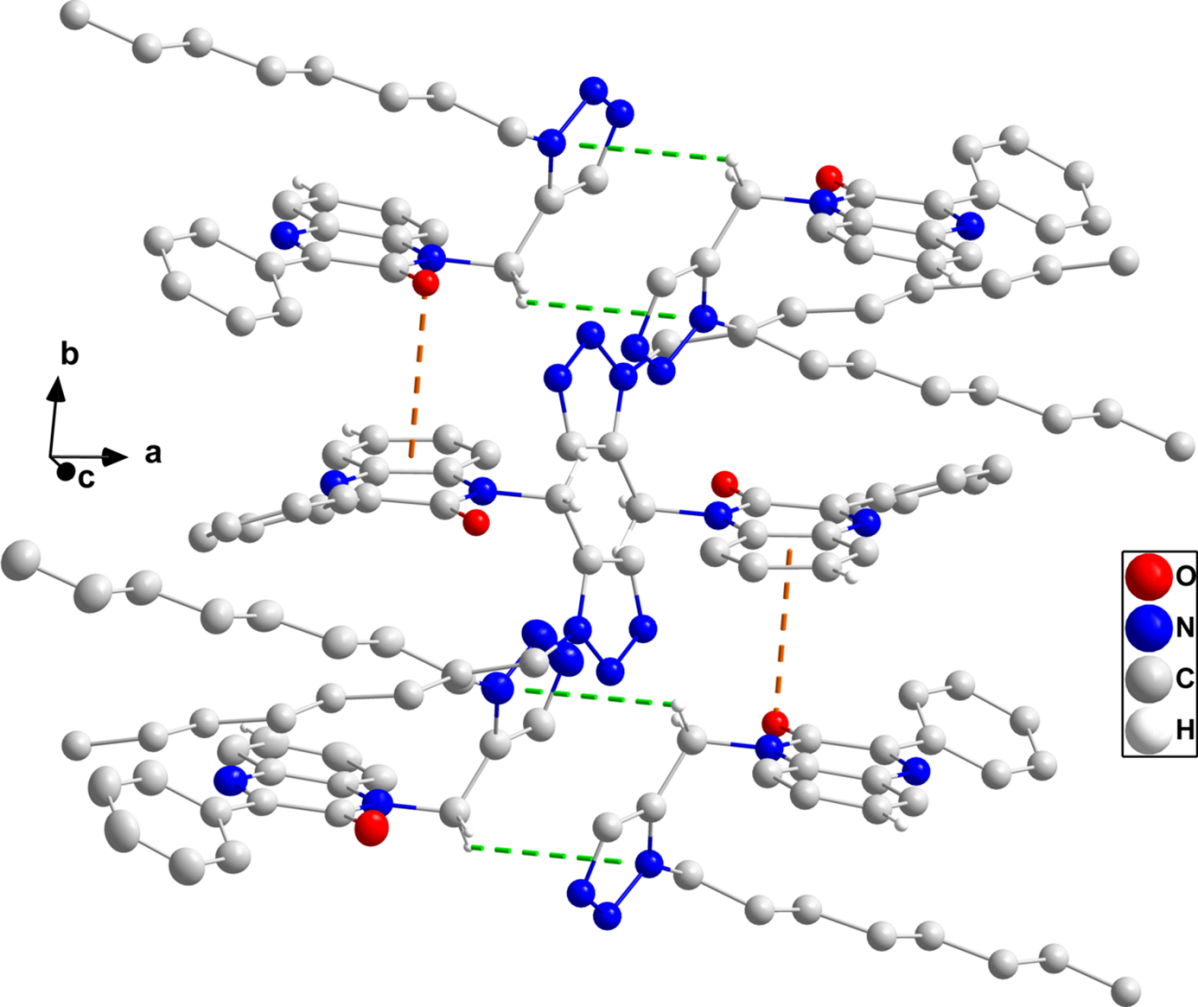
Detail of the inter­molecular C=O⋯π(ring) and C—H⋯π(ring) inter­actions, shown by orange and green dashed lines, respectively, with non-inter­acting hydrogen atoms omitted for clarity.

**Figure 4:**
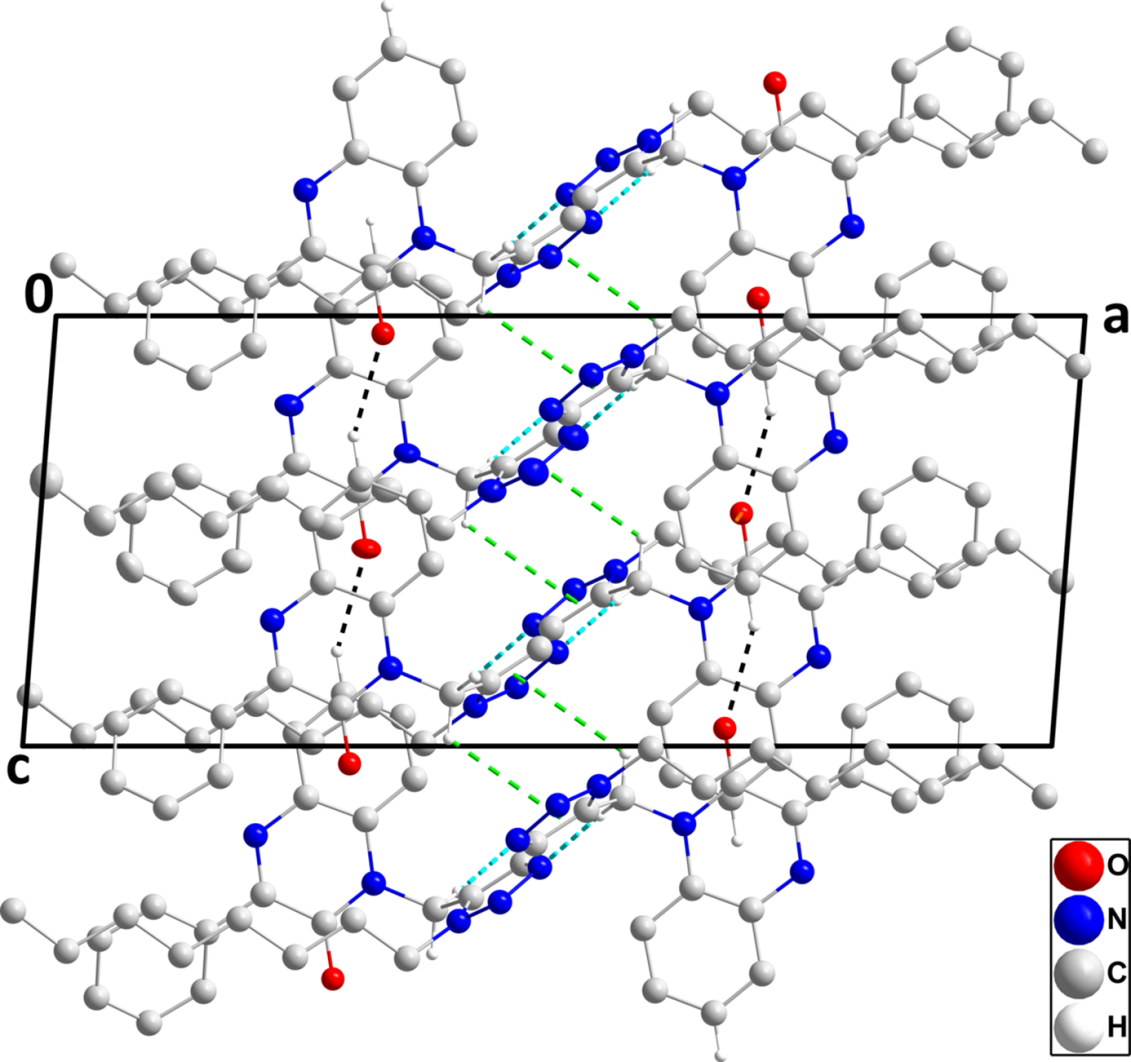
Packing viewed along the *b*-axis direction with inter­molecular inter­actions depicted as in Figs. 2 ▸ and 3 ▸ and with non-inter­acting hydrogen atoms omitted for clarity.

In the crystal, the polar portions of the mol­ecules (di­hydro­quinoxaline and triazole moieties) are associated through C3—H3⋯O1 and C15—H15*B*⋯N3 hydrogen bonds and C7=O1⋯*Cg*3 [*Cg*3 is the centroid of the C1–C6 ring at *x*, −*y* − 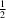, *z* − 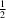 with C7⋯*Cg*3 = 3.542 (4) Å, O1⋯*Cg*3 = 3.397 (4) Å and C7=O1⋯*Cg*3 = 86.5 (2)°] and C15—H15*B*⋯*Cg*1 (*Cg*1 is the centroid of the triazole ring at −*x* + 1, −*y* + 1, −*z* + 1) inter­actions (Table 1 ▸), forming thick layers parallel to the *bc* plane. Fig. 2 ▸ shows a detail of the first three inter­molecular inter­actions while Fig. 3 ▸ illustrates the latter two inter­actions. Fig. 4 ▸ shows a portion of the full layer in which the hairpin loops of the *n*-octyl chains and the phenyl groups are on the outside surfaces. Consequently, the packing of the layers involves primarily van der Waals contacts between these groups.

## 4. Database survey

**Figure 5:**
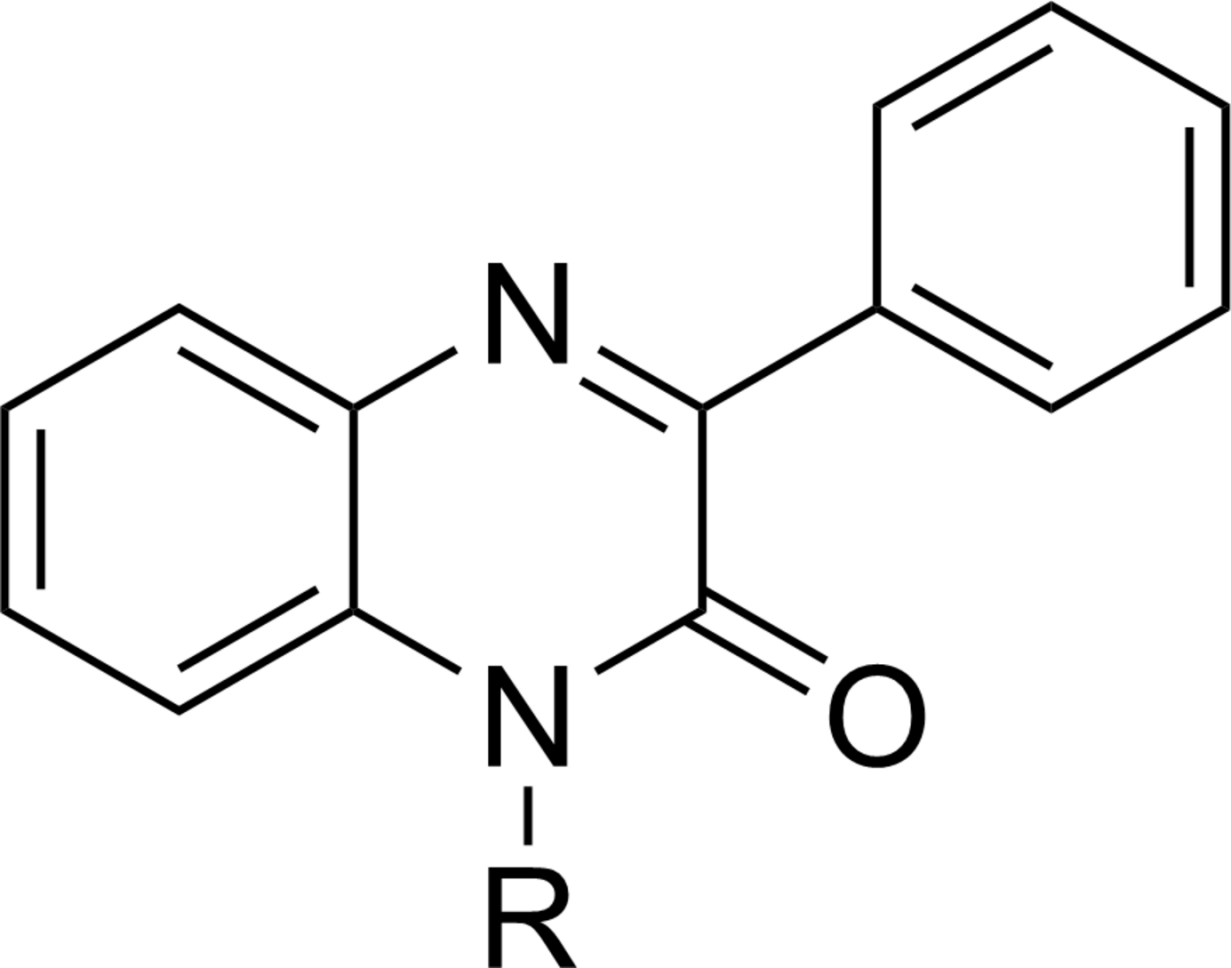
The fragment (*R* = C) used in the database search.

A search of the Cambridge Structural Database (CSD, updated to June 2024, Groom *et al.*, 2016 ▸) with the fragment shown in Fig. 5 ▸ (*R* = C) generated 29 hits of which 15 most resemble the title mol­ecule. These include those with *R* = Et (MAGBIJ; Al Ati *et al.*, 2021 ▸), Bz (PUGGII; Benzeid *et al.*, 2009 ▸), allyl (YAJGEX; Benzeid *et al.*, 2011 ▸), *n*-pentyl (UFIYEM; Abad *et al.*, 2023*b* ▸), *n*-octyl (AZAZEC; Abad *et al.*, 2023*a* ▸), *n*-nonyl (UDAMIZ; Abad *et al.*, 2021*a* ▸), CH_2_CO_2_Et (XEXWIJ; Abad *et al.*, 2018*a* ▸), CH_2_CH_2_CO_2_Et (ESUKUB; Abad *et al.*, 2021*b* ▸), CH_2_CH_2_CH_2_OH (RIRBOM; Abad *et al.*, 2018*b* ▸) and cyclo­propyl­methyl (NIBXEE; Abad *et al.*, 2018*c* ▸). More elaborate examples have *R* = (1-hex­yl)-1*H*-1,2,3-triazol-4-yl)methyl (FOFCIQ; Abad *et al.*, 2021*c* ▸), (1-ethyl­acetato)-1*H*-1,2,3-triazol-4-yl)methyl (ECUCOY; Abad *et al.*, 2022 ▸), (1,3-oxazolidin-2-one-3-yl)ethyl (IDOSUR; Daouda *et al.*, 2013 ▸) and (3-(*p*-tol­yl)-4,5-di­hydro­isoxazol-5-yl)methyl (ILIRED; Abad *et al.*, 2021*d* ▸). The two with the substituent on the ring nitro­gen of the di­hydro­quinoxaline that includes the 1,2,3-triazol-4-yl ring (ECUCOY and FOFCIQ) adopt comparable hairpin conformations. In the former, this results from an intra­molecular π-stacking inter­action between the two carbonyl groups, which are nearly anti-parallel to each other (centroid–centroid distance = 2.95 Å) while in the latter, there is an intra­molecular C—H⋯O hydrogen bond analogous to that in the title mol­ecule. A U-shaped conformation is adopted by IDOSUR but there is no intra­molecular inter­action with the side chain. In all the others, the substituent on the ring nitro­gen is in a largely extended conformation. In the examples cited, the di­hydro­qinoxaline moiety ranges from essentially planar (AZAZEC, ESUKUB, XEXWIJ and YAJGEX) to having a dihedral angle between the mean planes of the constituent rings as large as 4.51 (4)° (MAGBIJ). Additionally, the dihedral angle between the mean plane of the heterocyclic ring in the di­hydro­quinoxaline and that of the attached phenyl ring varies from 9.05 (7)° in ECUCOY to 43.61 (4)° in RIRBOM with the majority of them having this angle greater than 20°.

## 5. Hirshfeld surface analysis

**Figure 6:**
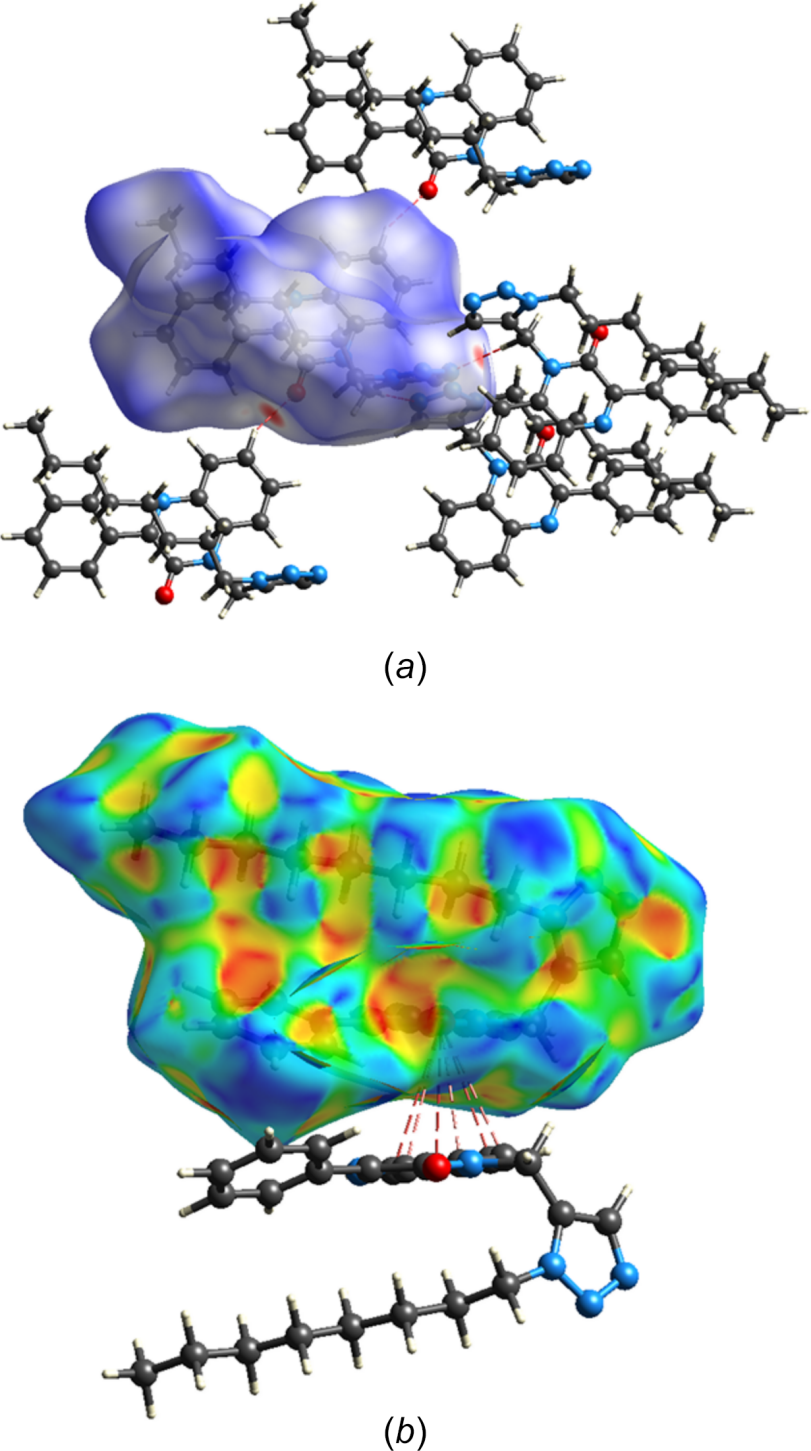
(*a*) The *d*_norm_ Hirshfeld surface showing the C—H⋯O and C—H⋯N hydrogen bonds to neighboring mol­ecules and (*b*) the surface calculated over shape function showing the C=O⋯π(ring) inter­action.

**Figure 7:**
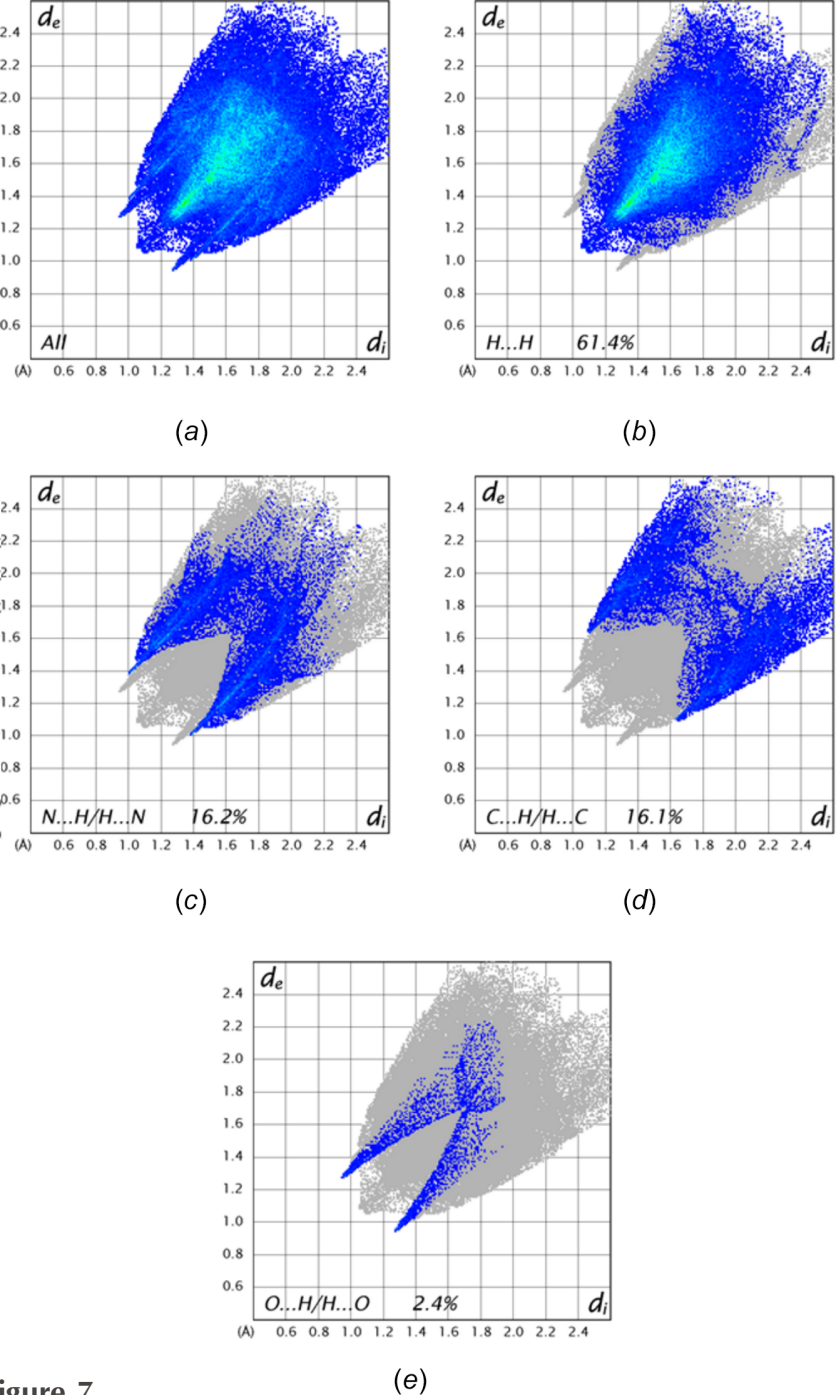
The 2-D fingerprint plots showing (*a*) all inter­molecular inter­actions and those delineated into (*b*) H⋯H, (*c*) N⋯H/H⋯N, (*d*) C⋯H/H⋯C and (*e*) O⋯H/H⋯O contacts. The percent contribution of each type is given in the Figure.

A Hirshfeld surface analysis was performed with *CrystalExplorer* (Spackman *et al.*, 2021 ▸) and the inter­pretation of the several plots obtained is described by Tan *et al.* (2019 ▸). Fig. 6 ▸*a* shows the *d*_norm_ surface together with four neighboring mol­ecules. Those above and below the surface show the C—H⋯O hydrogen bonds while those on the right show the C—H⋯N hydrogen bonds, which are also depicted in Fig. 2 ▸. Fig. 6 ▸*b* shows the surface calculated over the shape function with one neighboring mol­ecule illustrating the C7=O1⋯π(ring) inter­action. Fig. 7 ▸*a* is a 2-D fingerprint plot of all types of inter­molecular inter­actions with the remainder of the sections showing delineation into specific atom–atom contacts. The H⋯H contacts (Fig. 7 ▸*b*) contribute the lion’s share, which is not surprising considering the high hydrogen content, particularly in the *n*-octyl portion. These are followed by N⋯H/H⋯N (Fig. 7 ▸*c*), C⋯H/H⋯C (Fig. 7 ▸*d*) and O⋯H/H⋯O (Fig. 7 ▸*e*) contacts in order of decreasing percentage contribution. The N⋯H/H⋯N and O⋯H/H⋯O plots show rather sharp spikes as a result of the H⋯O and H⋯N distances having a narrow range of values since they primarily represent the C—H⋯O and C—H⋯N hydrogen bonds. All other contacts contribute considerably less, for example, the O⋯C contacts involving the C7=O1⋯π(ring) inter­actions contribute only 1.3% of the total.

## 6. Synthesis and crystallization

To a solution of 3-phenyl-1-(prop-2-yn-1-yl)quinoxalin-2(1*H*)-one 0.5 g (0.0020 mmol) in absolute ethanol (20 ml) were added 1.3 equivalents of 1-azido­octane. The mixture was stirred at reflux and the reaction monitored by thin layer chromatography (TLC). After concentration under reduced pressure, the residue was purified by column chromatography on silica gel using a mixture of ethyl acetate/hexane (10/90%) as eluent. The precipitated product was filtered off, dried and recrystallized from ethanol to yield colorless crystals of the title compound.

Yield 42%; m.p: 408–410 K; **^1^H NMR** (300 MHz, CDCl_3_) δ ppm: 0.90 (*t*, 3H, CH_3_, *J* = 6 Hz); 1.26–1.34 (*m*, 10H, CH_2_); 1.89 (*quin*, 2H, CH_2_); 4.58 (*t*, 2H, N—NCH, *J* = 6 Hz) ; 5.63 (*s*, 2H, N—CH_2_); 7.62 (*s*, 1H, CH_triazole_); 7.36-8.35 (*m*, 9H_arom_); **^13^C NMR** (75 MHz, CDCl_3_) δ ppm: 14.07 (CH_3_); 22.61, 26.12, 26.62, 29.06, 30.51, 31.69, 35.24 (CH_2_); 48.77 (N—CH); 113.38, 124.55, 128.21 (triazole); 129.54, 130.70, 130.74, 131.18, 131.27 (CH_arom_); 131.83, 133.47, 133.71, 135.53, 153.84 (Cq); 154.15 (C=O).

## 7. Refinement

Crystal data, data collection and structure refinement details are summarized in Table 2 ▸. H atoms were placed in calculated positions and included as riding contributions with isotropic displacement parameters tied to those of the attached atoms.

## Supplementary Material

Crystal structure: contains datablock(s) global, I. DOI: 10.1107/S2056989024007746/vm2306sup1.cif

Structure factors: contains datablock(s) I. DOI: 10.1107/S2056989024007746/vm2306Isup2.hkl

CCDC reference: 2061043

Additional supporting information:  crystallographic information; 3D view; checkCIF report

## Figures and Tables

**Table 1:** Hydrogen-bond geometry (Å, °) *Cg*1 and *Cg*2 are the centroids of the C16/C17/N3/N4/N5 and C1/C6/N1/C7/C8/N2 rings, respectively.

*D*—H⋯*A*	*D*—H	H⋯*A*	*D*⋯*A*	*D*—H⋯*A*
C3—H3⋯O1^i^	0.95	2.34	3.225 (5)	155
C15—H15*A*⋯N3^ii^	0.99	2.48	3.467 (5)	174
C15—H15*B*⋯*Cg*1^iii^	0.99	2.96	3.575 (4)	121
C18—H18*B*⋯O1	0.99	2.47	3.291 (5)	140
C19—H19*A*⋯*Cg*2	0.99	2.65	3.646 (4)	148

Symmetry codes: (i) 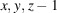; (ii) 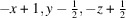; (iii) 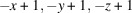.

**Table 2:** Experimental details

Crystal data	
Chemical formula	C_25_H_29_N_5_O
*M*_r_	415.53
Crystal system, space group	Monoclinic, *P*2_1_/*c*
Temperature (K)	150
*a*, *b*, *c* (Å)	22.5143 (9), 10.5927 (4), 9.4449 (3)
β (°)	94.439 (2)
*V* (Å^3^)	2245.73 (14)
*Z*	4
Radiation type	Cu *K*α
μ (mm^−1^)	0.61
Crystal size (mm)	0.22 × 0.14 × 0.03

Data collection	
Diffractometer	Bruker D8 VENTURE PHOTON 100 CMOS
Absorption correction	Multi-scan (*SADABS*; Krause *et al.*, 2015 ▸)
*T*_min_, *T*_max_	0.78, 0.98
No. of measured, independent and observed [*I* > 2σ(*I*)] reflections	14897, 4077, 2414
*R*_int_	0.100
(sin θ/λ)_max_ (Å^−1^)	0.603

Refinement	
*R*[*F*^2^ > 2σ(*F*^2^)], *wR*(*F*^2^), *S*	0.089, 0.187, 1.08
No. of reflections	4077
No. of parameters	281
H-atom treatment	H-atom parameters constrained
Δρ_max_, Δρ_min_ (e Å^−3^)	0.29, −0.25

Computer programs: *APEX3* and *SAINT* (Bruker, 2016 ▸), *SHELXT* (Sheldrick, 2015*a* ▸), *SHELXL2018/1* (Sheldrick, 2015*b* ▸), *DIAMOND* (Brandenburg & Putz, 2012 ▸) and *SHELXTL* (Sheldrick, 2008 ▸).

## supplementary crystallographic information

### 1-[(1-Octyl-1H-1,2,3-triazol-4-yl)methyl]-3-phenyl-1,2-dihydroquinoxalin-2(1H)-one Crystal data

**Table:**

C_25_H_29_N_5_O	*F*(000) = 888
*M**_r_* = 415.53	*D*_x_ = 1.229 Mg m^−^^3^
Monoclinic, *P*2_1_/*c*	Cu *K*α radiation, λ = 1.54178 Å
*a* = 22.5143 (9) Å	Cell parameters from 6427 reflections
*b* = 10.5927 (4) Å	θ = 3.9–68.3°
*c* = 9.4449 (3) Å	µ = 0.61 mm^−^^1^
β = 94.439 (2)°	*T* = 150 K
*V* = 2245.73 (14) Å^3^	Plate, colourless
*Z* = 4	0.22 × 0.14 × 0.03 mm

### 1-[(1-Octyl-1H-1,2,3-triazol-4-yl)methyl]-3-phenyl-1,2-dihydroquinoxalin-2(1H)-one Data collection

**Table:**

Bruker D8 VENTURE PHOTON 100 CMOS diffractometer	4077 independent reflections
Radiation source: INCOATEC IµS micro–focus source	2414 reflections with *I* > 2σ(*I*)
Mirror monochromator	*R*_int_ = 0.100
ω scans	θ_max_ = 68.4°, θ_min_ = 2.0°
Absorption correction: multi-scan (*SADABS*; Krause *et al.*, 2015)	*h* = −24→26
*T*_min_ = 0.78, *T*_max_ = 0.98	*k* = −12→12
14897 measured reflections	*l* = −11→11

### 1-[(1-Octyl-1H-1,2,3-triazol-4-yl)methyl]-3-phenyl-1,2-dihydroquinoxalin-2(1H)-one Refinement

**Table:**

Refinement on *F*^2^	Primary atom site location: structure-invariant direct methods
Least-squares matrix: full	Secondary atom site location: difference Fourier map
*R*[*F*^2^ > 2σ(*F*^2^)] = 0.089	Hydrogen site location: inferred from neighbouring sites
*wR*(*F*^2^) = 0.187	H-atom parameters constrained
*S* = 1.08	*w* = 1/[σ^2^(*F*_o_^2^) + (0.0212*P*)^2^ + 4.325*P*] where *P* = (*F*_o_^2^ + 2*F*_c_^2^)/3
4077 reflections	(Δ/σ)_max_ < 0.001
281 parameters	Δρ_max_ = 0.28 e Å^−^^3^
0 restraints	Δρ_min_ = −0.25 e Å^−^^3^

### 1-[(1-Octyl-1H-1,2,3-triazol-4-yl)methyl]-3-phenyl-1,2-dihydroquinoxalin-2(1H)-one Special details

**Table:**

Geometry. All esds (except the esd in the dihedral angle between two l.s. planes) are estimated using the full covariance matrix. The cell esds are taken into account individually in the estimation of esds in distances, angles and torsion angles; correlations between esds in cell parameters are only used when they are defined by crystal symmetry. An approximate (isotropic) treatment of cell esds is used for estimating esds involving l.s. planes.
Refinement. Refinement of F^2^ against ALL reflections. The weighted R-factor wR and goodness of fit S are based on F^2^, conventional R-factors R are based on F, with F set to zero for negative F^2^. The threshold expression of F^2^ > 2sigma(F^2^) is used only for calculating R-factors(gt) etc. and is not relevant to the choice of reflections for refinement. R-factors based on F^2^ are statistically about twice as large as those based on F, and R- factors based on ALL data will be even larger. H-atoms attached to carbon were placed in calculated positions (C—H = 0.95 - 0.99 Å) and included as riding contributions with isotropic displacement parameters 1.2 - 1.5 times those of the attached atoms.

### 1-[(1-Octyl-1H-1,2,3-triazol-4-yl)methyl]-3-phenyl-1,2-dihydroquinoxalin-2(1H)-one Fractional atomic coordinates and isotropic or equivalent isotropic displacement parameters (Å^2^)

**Table:**

	*x*	*y*	*z*	*U*_iso_*/*U*_eq_	
O1	0.31905 (12)	0.4116 (3)	0.5411 (3)	0.0402 (7)	
N1	0.35153 (14)	0.4054 (3)	0.3191 (3)	0.0304 (7)	
N2	0.23311 (15)	0.4198 (3)	0.2078 (3)	0.0351 (8)	
N3	0.51179 (16)	0.6643 (3)	0.2817 (4)	0.0450 (9)	
N4	0.47557 (16)	0.7245 (3)	0.3627 (4)	0.0442 (9)	
N5	0.43688 (15)	0.6386 (3)	0.4067 (3)	0.0375 (8)	
C1	0.27946 (17)	0.4162 (3)	0.1195 (4)	0.0312 (8)	
C2	0.26428 (19)	0.4209 (4)	−0.0285 (4)	0.0374 (9)	
H2	0.223790	0.428795	−0.063874	0.045*	
C3	0.3081 (2)	0.4141 (4)	−0.1205 (4)	0.0415 (10)	
H3	0.298302	0.418698	−0.219991	0.050*	
C4	0.3670 (2)	0.4003 (4)	−0.0680 (4)	0.0408 (10)	
H4	0.397071	0.394595	−0.132888	0.049*	
C5	0.38320 (19)	0.3946 (3)	0.0766 (4)	0.0356 (9)	
H5	0.423674	0.383697	0.110762	0.043*	
C6	0.33873 (17)	0.4054 (3)	0.1702 (4)	0.0296 (8)	
C7	0.30655 (17)	0.4091 (3)	0.4117 (4)	0.0296 (8)	
C8	0.24497 (17)	0.4129 (3)	0.3448 (4)	0.0319 (8)	
C9	0.19308 (17)	0.4142 (4)	0.4323 (4)	0.0337 (9)	
C10	0.13926 (19)	0.4627 (4)	0.3735 (5)	0.0435 (10)	
H10	0.137071	0.495971	0.279822	0.052*	
C11	0.0893 (2)	0.4635 (5)	0.4480 (5)	0.0509 (12)	
H11	0.053282	0.498797	0.406174	0.061*	
C12	0.0908 (2)	0.4131 (5)	0.5841 (5)	0.0527 (12)	
H12	0.056176	0.413158	0.635478	0.063*	
C13	0.1439 (2)	0.3626 (4)	0.6434 (4)	0.0461 (11)	
H13	0.145473	0.327016	0.735990	0.055*	
C14	0.1942 (2)	0.3636 (4)	0.5695 (4)	0.0412 (10)	
H14	0.230287	0.329379	0.612140	0.049*	
C15	0.41335 (17)	0.4045 (4)	0.3792 (4)	0.0339 (9)	
H15A	0.434130	0.331865	0.339415	0.041*	
H15B	0.413718	0.391802	0.483141	0.041*	
C16	0.44727 (18)	0.5225 (4)	0.3517 (4)	0.0342 (9)	
C17	0.49494 (18)	0.5414 (4)	0.2736 (4)	0.0392 (10)	
H17	0.513561	0.477949	0.221367	0.047*	
C18	0.38948 (18)	0.6787 (4)	0.4936 (4)	0.0405 (10)	
H18A	0.404178	0.748811	0.556118	0.049*	
H18B	0.378803	0.607611	0.554828	0.049*	
C19	0.33455 (18)	0.7215 (4)	0.4043 (4)	0.0403 (10)	
H19A	0.324586	0.657968	0.329307	0.048*	
H19B	0.343389	0.802013	0.357084	0.048*	
C20	0.28082 (18)	0.7401 (4)	0.4907 (4)	0.0415 (10)	
H20A	0.275342	0.663014	0.547534	0.050*	
H20B	0.289133	0.810885	0.557656	0.050*	
C21	0.22352 (19)	0.7672 (4)	0.4019 (4)	0.0426 (10)	
H21A	0.229096	0.844375	0.345237	0.051*	
H21B	0.215452	0.696557	0.334558	0.051*	
C22	0.16970 (18)	0.7854 (4)	0.4858 (4)	0.0435 (10)	
H22A	0.163263	0.707438	0.540295	0.052*	
H22B	0.178002	0.854695	0.554860	0.052*	
C23	0.11349 (19)	0.8158 (4)	0.3957 (5)	0.0480 (11)	
H23A	0.107317	0.750420	0.321071	0.058*	
H23B	0.118883	0.897606	0.347650	0.058*	
C24	0.0581 (2)	0.8234 (5)	0.4759 (5)	0.0544 (12)	
H24A	0.064869	0.885171	0.554164	0.065*	
H24B	0.050943	0.740002	0.518780	0.065*	
C25	0.0031 (2)	0.8620 (5)	0.3828 (6)	0.0647 (14)	
H25A	−0.031573	0.862778	0.439565	0.097*	
H25B	−0.003742	0.801635	0.304585	0.097*	
H25C	0.008978	0.946546	0.344113	0.097*	

### 1-[(1-Octyl-1H-1,2,3-triazol-4-yl)methyl]-3-phenyl-1,2-dihydroquinoxalin-2(1H)-one Atomic displacement parameters (Å^2^)

**Table:**

	*U*^11^	*U*^22^	*U*^33^	*U*^12^	*U*^13^	*U*^23^
O1	0.0462 (17)	0.0528 (17)	0.0217 (13)	0.0000 (14)	0.0040 (11)	0.0017 (12)
N1	0.0373 (19)	0.0333 (17)	0.0211 (15)	0.0002 (14)	0.0044 (13)	0.0013 (13)
N2	0.044 (2)	0.0360 (18)	0.0260 (16)	0.0005 (15)	0.0054 (14)	−0.0007 (14)
N3	0.049 (2)	0.048 (2)	0.039 (2)	−0.0081 (17)	0.0083 (17)	−0.0018 (17)
N4	0.049 (2)	0.042 (2)	0.042 (2)	−0.0085 (17)	0.0065 (17)	0.0006 (17)
N5	0.043 (2)	0.0406 (19)	0.0292 (17)	−0.0027 (16)	0.0029 (15)	−0.0005 (15)
C1	0.038 (2)	0.0289 (19)	0.0274 (18)	0.0015 (17)	0.0037 (16)	−0.0018 (16)
C2	0.045 (3)	0.040 (2)	0.0262 (19)	0.0018 (19)	−0.0045 (17)	0.0017 (17)
C3	0.062 (3)	0.042 (2)	0.0214 (19)	−0.002 (2)	0.0072 (19)	−0.0020 (17)
C4	0.058 (3)	0.034 (2)	0.032 (2)	−0.004 (2)	0.016 (2)	−0.0036 (17)
C5	0.048 (3)	0.031 (2)	0.0293 (19)	0.0001 (18)	0.0080 (17)	−0.0033 (16)
C6	0.039 (2)	0.0281 (19)	0.0214 (17)	0.0019 (16)	0.0028 (16)	0.0008 (15)
C7	0.036 (2)	0.0310 (19)	0.0217 (18)	0.0030 (16)	0.0015 (15)	−0.0006 (15)
C8	0.038 (2)	0.0292 (19)	0.0283 (19)	0.0007 (17)	0.0015 (16)	−0.0007 (16)
C9	0.038 (2)	0.036 (2)	0.0282 (19)	−0.0005 (18)	0.0052 (17)	−0.0020 (17)
C10	0.047 (3)	0.047 (2)	0.037 (2)	0.000 (2)	0.006 (2)	−0.005 (2)
C11	0.039 (3)	0.062 (3)	0.051 (3)	0.004 (2)	0.005 (2)	−0.003 (2)
C12	0.041 (3)	0.067 (3)	0.052 (3)	−0.002 (2)	0.017 (2)	−0.007 (2)
C13	0.048 (3)	0.056 (3)	0.036 (2)	−0.004 (2)	0.012 (2)	0.001 (2)
C14	0.048 (3)	0.044 (2)	0.033 (2)	0.000 (2)	0.0063 (19)	0.0016 (19)
C15	0.036 (2)	0.036 (2)	0.0308 (19)	0.0022 (17)	0.0046 (17)	−0.0013 (17)
C16	0.039 (2)	0.037 (2)	0.0272 (19)	0.0009 (18)	0.0025 (17)	−0.0001 (17)
C17	0.038 (2)	0.043 (2)	0.037 (2)	−0.0027 (18)	0.0057 (18)	−0.0021 (19)
C18	0.044 (3)	0.044 (2)	0.033 (2)	0.000 (2)	0.0059 (18)	−0.0046 (19)
C19	0.049 (3)	0.038 (2)	0.034 (2)	0.0016 (19)	0.0006 (19)	−0.0016 (18)
C20	0.048 (3)	0.042 (2)	0.035 (2)	0.005 (2)	0.0049 (19)	−0.0033 (19)
C21	0.054 (3)	0.038 (2)	0.035 (2)	0.003 (2)	0.003 (2)	−0.0026 (18)
C22	0.043 (3)	0.048 (3)	0.039 (2)	0.003 (2)	0.0028 (19)	−0.002 (2)
C23	0.052 (3)	0.049 (3)	0.043 (3)	0.006 (2)	0.001 (2)	0.003 (2)
C24	0.054 (3)	0.059 (3)	0.051 (3)	0.011 (2)	0.007 (2)	−0.001 (2)
C25	0.057 (3)	0.067 (3)	0.071 (4)	0.009 (3)	0.004 (3)	0.005 (3)

### 1-[(1-Octyl-1H-1,2,3-triazol-4-yl)methyl]-3-phenyl-1,2-dihydroquinoxalin-2(1H)-one Geometric parameters (Å, º)

**Table:**

O1—C7	1.233 (4)	C13—H13	0.9500
N1—C7	1.389 (4)	C14—H14	0.9500
N1—C6	1.413 (4)	C15—C16	1.498 (5)
N1—C15	1.462 (5)	C15—H15A	0.9900
N2—C8	1.303 (5)	C15—H15B	0.9900
N2—C1	1.386 (5)	C16—C17	1.363 (5)
N3—N4	1.324 (5)	C17—H17	0.9500
N3—C17	1.357 (5)	C18—C19	1.512 (6)
N4—N5	1.347 (4)	C18—H18A	0.9900
N5—C16	1.362 (5)	C18—H18B	0.9900
N5—C18	1.459 (5)	C19—C20	1.523 (5)
C1—C6	1.387 (5)	C19—H19A	0.9900
C1—C2	1.415 (5)	C19—H19B	0.9900
C2—C3	1.366 (5)	C20—C21	1.511 (5)
C2—H2	0.9500	C20—H20A	0.9900
C3—C4	1.386 (6)	C20—H20B	0.9900
C3—H3	0.9500	C21—C22	1.511 (5)
C4—C5	1.387 (5)	C21—H21A	0.9900
C4—H4	0.9500	C21—H21B	0.9900
C5—C6	1.391 (5)	C22—C23	1.504 (6)
C5—H5	0.9500	C22—H22A	0.9900
C7—C8	1.479 (5)	C22—H22B	0.9900
C8—C9	1.483 (5)	C23—C24	1.510 (6)
C9—C10	1.392 (6)	C23—H23A	0.9900
C9—C14	1.400 (5)	C23—H23B	0.9900
C10—C11	1.373 (6)	C24—C25	1.520 (6)
C10—H10	0.9500	C24—H24A	0.9900
C11—C12	1.390 (6)	C24—H24B	0.9900
C11—H11	0.9500	C25—H25A	0.9800
C12—C13	1.387 (6)	C25—H25B	0.9800
C12—H12	0.9500	C25—H25C	0.9800
C13—C14	1.377 (6)		
			
C7—N1—C6	121.6 (3)	H15A—C15—H15B	107.6
C7—N1—C15	118.3 (3)	N5—C16—C17	103.9 (3)
C6—N1—C15	120.1 (3)	N5—C16—C15	125.6 (3)
C8—N2—C1	119.3 (3)	C17—C16—C15	130.5 (4)
N4—N3—C17	108.2 (3)	N3—C17—C16	109.8 (4)
N3—N4—N5	107.2 (3)	N3—C17—H17	125.1
N4—N5—C16	111.0 (3)	C16—C17—H17	125.1
N4—N5—C18	119.7 (3)	N5—C18—C19	112.1 (3)
C16—N5—C18	129.2 (3)	N5—C18—H18A	109.2
N2—C1—C6	122.9 (3)	C19—C18—H18A	109.2
N2—C1—C2	117.3 (3)	N5—C18—H18B	109.2
C6—C1—C2	119.8 (3)	C19—C18—H18B	109.2
C3—C2—C1	119.7 (4)	H18A—C18—H18B	107.9
C3—C2—H2	120.1	C18—C19—C20	112.9 (3)
C1—C2—H2	120.1	C18—C19—H19A	109.0
C2—C3—C4	119.7 (4)	C20—C19—H19A	109.0
C2—C3—H3	120.1	C18—C19—H19B	109.0
C4—C3—H3	120.1	C20—C19—H19B	109.0
C3—C4—C5	121.9 (4)	H19A—C19—H19B	107.8
C3—C4—H4	119.1	C21—C20—C19	114.0 (3)
C5—C4—H4	119.1	C21—C20—H20A	108.7
C4—C5—C6	118.3 (4)	C19—C20—H20A	108.7
C4—C5—H5	120.8	C21—C20—H20B	108.7
C6—C5—H5	120.8	C19—C20—H20B	108.7
C1—C6—C5	120.5 (3)	H20A—C20—H20B	107.6
C1—C6—N1	117.4 (3)	C22—C21—C20	114.7 (3)
C5—C6—N1	122.1 (4)	C22—C21—H21A	108.6
O1—C7—N1	120.2 (3)	C20—C21—H21A	108.6
O1—C7—C8	123.9 (3)	C22—C21—H21B	108.6
N1—C7—C8	115.9 (3)	C20—C21—H21B	108.6
N2—C8—C7	122.6 (3)	H21A—C21—H21B	107.6
N2—C8—C9	116.3 (4)	C23—C22—C21	113.9 (4)
C7—C8—C9	121.0 (3)	C23—C22—H22A	108.8
C10—C9—C14	117.6 (4)	C21—C22—H22A	108.8
C10—C9—C8	118.7 (3)	C23—C22—H22B	108.8
C14—C9—C8	123.6 (4)	C21—C22—H22B	108.8
C11—C10—C9	121.4 (4)	H22A—C22—H22B	107.7
C11—C10—H10	119.3	C22—C23—C24	114.7 (4)
C9—C10—H10	119.3	C22—C23—H23A	108.6
C10—C11—C12	120.6 (5)	C24—C23—H23A	108.6
C10—C11—H11	119.7	C22—C23—H23B	108.6
C12—C11—H11	119.7	C24—C23—H23B	108.6
C13—C12—C11	118.8 (4)	H23A—C23—H23B	107.6
C13—C12—H12	120.6	C23—C24—C25	113.1 (4)
C11—C12—H12	120.6	C23—C24—H24A	109.0
C14—C13—C12	120.6 (4)	C25—C24—H24A	109.0
C14—C13—H13	119.7	C23—C24—H24B	109.0
C12—C13—H13	119.7	C25—C24—H24B	109.0
C13—C14—C9	121.0 (4)	H24A—C24—H24B	107.8
C13—C14—H14	119.5	C24—C25—H25A	109.5
C9—C14—H14	119.5	C24—C25—H25B	109.5
N1—C15—C16	114.2 (3)	H25A—C25—H25B	109.5
N1—C15—H15A	108.7	C24—C25—H25C	109.5
C16—C15—H15A	108.7	H25A—C25—H25C	109.5
N1—C15—H15B	108.7	H25B—C25—H25C	109.5
C16—C15—H15B	108.7		
			
C17—N3—N4—N5	1.0 (5)	C7—C8—C9—C10	156.9 (4)
N3—N4—N5—C16	−1.2 (4)	N2—C8—C9—C14	156.1 (4)
N3—N4—N5—C18	−177.4 (3)	C7—C8—C9—C14	−26.3 (6)
C8—N2—C1—C6	−0.1 (6)	C14—C9—C10—C11	1.3 (6)
C8—N2—C1—C2	178.5 (3)	C8—C9—C10—C11	178.3 (4)
N2—C1—C2—C3	−178.2 (4)	C9—C10—C11—C12	−1.4 (7)
C6—C1—C2—C3	0.4 (6)	C10—C11—C12—C13	0.3 (7)
C1—C2—C3—C4	1.1 (6)	C11—C12—C13—C14	0.6 (7)
C2—C3—C4—C5	−0.8 (6)	C12—C13—C14—C9	−0.6 (7)
C3—C4—C5—C6	−1.1 (6)	C10—C9—C14—C13	−0.4 (6)
N2—C1—C6—C5	176.2 (4)	C8—C9—C14—C13	−177.1 (4)
C2—C1—C6—C5	−2.3 (6)	C7—N1—C15—C16	−111.7 (4)
N2—C1—C6—N1	−3.6 (6)	C6—N1—C15—C16	66.8 (4)
C2—C1—C6—N1	177.9 (3)	N4—N5—C16—C17	1.0 (4)
C4—C5—C6—C1	2.7 (6)	C18—N5—C16—C17	176.6 (4)
C4—C5—C6—N1	−177.6 (3)	N4—N5—C16—C15	179.3 (4)
C7—N1—C6—C1	3.4 (5)	C18—N5—C16—C15	−5.1 (7)
C15—N1—C6—C1	−175.1 (3)	N1—C15—C16—N5	67.6 (5)
C7—N1—C6—C5	−176.4 (4)	N1—C15—C16—C17	−114.6 (5)
C15—N1—C6—C5	5.2 (5)	N4—N3—C17—C16	−0.4 (5)
C6—N1—C7—O1	−178.5 (3)	N5—C16—C17—N3	−0.3 (5)
C15—N1—C7—O1	−0.1 (5)	C15—C16—C17—N3	−178.5 (4)
C6—N1—C7—C8	0.1 (5)	N4—N5—C18—C19	87.1 (4)
C15—N1—C7—C8	178.6 (3)	C16—N5—C18—C19	−88.2 (5)
C1—N2—C8—C7	3.9 (6)	N5—C18—C19—C20	169.2 (4)
C1—N2—C8—C9	−178.5 (3)	C18—C19—C20—C21	−172.9 (4)
O1—C7—C8—N2	174.7 (4)	C19—C20—C21—C22	179.8 (4)
N1—C7—C8—N2	−3.9 (5)	C20—C21—C22—C23	178.5 (4)
O1—C7—C8—C9	−2.7 (6)	C21—C22—C23—C24	175.0 (4)
N1—C7—C8—C9	178.6 (3)	C22—C23—C24—C25	176.5 (4)
N2—C8—C9—C10	−20.7 (6)		

### 1-[(1-Octyl-1H-1,2,3-triazol-4-yl)methyl]-3-phenyl-1,2-dihydroquinoxalin-2(1H)-one Hydrogen-bond geometry (Å, º)

*Cg*1 and *Cg*2 are the centroids of the C16/C17/N3/N4/N5 and C1/C6/N1/C7/C8/N2 rings, respectively.

**Table:**

*D*—H···*A*	*D*—H	H···*A*	*D*···*A*	*D*—H···*A*
C3—H3···O1^i^	0.95	2.34	3.225 (5)	155
C15—H15*A*···N3^ii^	0.99	2.48	3.467 (5)	174
C15—H15*B*···*Cg*1^iii^	0.99	2.96	3.575 (4)	121
C18—H18*B*···O1	0.99	2.47	3.291 (5)	140
C19—H19*A*···*Cg*2	0.99	2.65	3.646 (4)	148

Symmetry codes: (i) *x*, *y*, *z*−1; (ii) −*x*+1, *y*−1/2, −*z*+1/2; (iii) −*x*+1, −*y*+1, −*z*+1.
